# Investigation of bioactivity of unsaturated oligo‑galacturonic acids produced from apple waste by *Alcaligenes faecalis* AGS3 and *Paenibacillus polymyxa* S4 Pectinases

**DOI:** 10.1038/s41598-022-20011-2

**Published:** 2022-09-22

**Authors:** Behnam Ashrafian, Afrouzossadat Hosseini-Abari

**Affiliations:** Department of Cell and Molecular Biology & Microbiology, Faculty of Biological Science and Technology, University of Isfahan, Isfahan, 8174673441 Iran

**Keywords:** Biotechnology, Microbiology

## Abstract

Pectin is one of the main structural components in fruits and an indigestible fiber made of d-galacturonic acid units with α (1-4) linkage. This study investigates the microbial degradation of pectin in apple waste and the production of bioactive compounds. Firstly, pectin-degrading bacteria were isolated and identified, then pectinolytic activity was assessed by DNS. The products were evaluated by TLC and LC–MS–ESI. The antioxidative effects were investigated using DPPH and anti-cancer effects and cytotoxicity were analyzed by MTT and flow cytometry. In this study two new bacterial isolates, *Alcaligenes faecalis* AGS3 and *Paenibacillus polymyxa* S4 with the pectinolytic enzyme were introduced. Structure analysis showed that the products of enzymatic degradation include unsaturated mono, di, tri, and penta galacturonic acids with 74% and 69% RSA at 40 mg/mL for *A. faecalis* and *P. polymyxa* S4, respectively. The results of anti-tumor properties on MCF-7 cells by MTT assay, for products of AGS3 and S4 at 40 mg/mL after 48 h, showed 7% and 9% survival, respectively. In the flow cytometric assessment, the compounds of AGS3 at 40 mg/mL were 100% lethal in 48 h and regarding S4 isolate caused 98% death. Cytotoxicity evaluation on L-929 cells showed no significant toxicity on living cells.

## Introduction

As the population of Earth grows, increment of food production to meet the nutritional needs has always been one of the main concerns of human societies. This increase in production, along with factors such as uncontrolled urbanization and the lack of appropriate waste management and recycling methods, leads to the accumulation of waste in the environment, which causes irreversible environmental damages^1^. According to the Food and Agriculture Organization of the United Nations (FAO), the production of fruits and vegetables in the world is more than 1.74 billion tons, of which 10–50 percent of it is wasted in different countries. The value of food waste in the world is estimated at US$ 1 trillion annually. The resources used to produce that amount of wasted food are responsible for the emission of 4.4 gigatons of greenhouse gases (equivalent to CO_2_) per year, making wasted food the third-largest producer of greenhouse gases in the world after China and the United States^2^.

The extent of food losses varies at different stages of the production chain depending on the type of product, the level of economic development, and the social and cultural conditions in a geographical area. In the case of fruits and vegetables, according to the FAO study, wastage in the harvesting, sorting, and grading stages is predominant in industrial areas. In developing areas, while food loss is high during the harvesting, sorting, and grading stages, the amount of wastage during the processing stage (14–21%) is much higher than in developed areas (less than 2%)^2,3^. This is while most fruit products are processed before consumption, so in addition to increasing the value, also maintains the quality and number of fruits on the market^4^. Large amounts of waste are accumulated during the processing, which imposes high costs for treatments to reduce environmental detriment^5,6^. Thus, fruit processing waste not only accounts for significant amounts of food waste, which means serious environmental damage but also indicates the loss of nutrients of high value^7^. Therefore, the conversion of waste into value-added products is important and necessary to improve the sustainability and efficiency of the food supply chain^8^.

Over the years, in numerous studies, the production of value-added products, through microbial conversion or enzymatic processes of fruit wastes such as enzymes, bioethanol, organic acids, heteropolysaccharides, aromatic compounds, protein-fortified feed, prebiotic oligosaccharides, and bioactive compounds, has been investigated^9^. Microbial processing of residual fruit fibers is a relatively new approach that uses waste as monomers for the synthesis of natural beneficial oligomers^10^.

In general, the structure of the fruit is composed of various compounds with different properties. After water, the highest abundance belongs to carbohydrates, which make up 50 to 80% of the dry weight in most fruits^11^. A large number of carbohydrates in fruit waste are types of dietary fiber, of which pectin makes up to 40% of the compounds^12^. Pectins are a family of high molecular weight acidic polysaccharides, which are abundant in the primary cell wall of plants, made primarily from d-galacturonic acid units in the main chain with α (1-4) linkage along with small amounts of rhamnose in the main chain and arabinose, galactose and xylose in the side chains^10,13^. Several studies have demonstrated the applications of pectin in the food and pharmaceutical industries. Studies have suggested that short-chain pectins have wider applications, such as prebiotic, anti-cancer agents, drug delivery systems, radical scavenging properties, cholesterol-lowering, etc., and those features are related to the structure and molecular weight of pectic compounds^10,14^.

Radical species are associated with chronic obstructive pulmonary disease (COPD), asthma, diabetes, inflammation, cardiovascular disease, and myocardial infarction. Synthetic antioxidants commonly used in manufacturing processes are considered to be the cause of cytotoxicity. On the other hand, natural polysaccharides are considered to be reliable antioxidants that can scavenge free radicals and overcome synthetic substances that pose health concerns. The compounds rich in rhamnogalacturonan I as well as the compounds consisting of homogalacturonan in the main chain and rhamnogalacturonan I and arabinogalactan in the side chains show the highest level of antioxidant properties; Therefore, the antioxidant properties of pectic compounds can be traced to the structure of rhamnogalacturonan I, homogalacturonan and arabinogalactan^15^. Oligogalacturonic acids derived from pectin, modified by various treatments also can purify compounds including anion superoxide, hydroxyl, and reactive oxygen species. They also increase the antioxidant properties of enzymes: superoxide dismutase, catalase, and glutathione peroxidase and show antioxidant properties by increasing the amount of glutathione^16^.

Another promising biological activity of pectin is its potential role in preventing and reducing carcinogenesis. The positive effects of “modified pectin”, whether in the form of commercial products such as modified citrus pectin (e.g. GCS-100, FPP, or pectasol) or laboratory-modified pectin, have been studied and proven. Citrus modified pectin (MCP) in particular has been and is under extensive research^17^. Not all MCPs are the same because the term is used to describe water-soluble polysaccharides derived from pectin produced from citrus peel and pulp treated with high temperature, high pH, or enzymatic decomposition. pH-modified citrus pectin has reduced molecular weight and is derived from commercial citrus pectin and has galactose-rich side chains that are capable of binding to the prometastatic protein galactin-3 and inhibiting tumor cell metastasis. Galactin-3 plays a key role in several intracellular physiological and pathological processes, including tumor cell proliferation and apoptosis. The binding of MCP to galactin-3 may inhibit the negative effects of galactin-3, such as inhibiting its ability to promote adhesion and migration of tumor cells and prevent apoptosis. In addition to galactose-rich side chains, the homogalacturonan part of pectin also has anti-cancer activity. Low molecular weight pectin fragments (1 kDa) rich in galacturonic acid were absorbed by mouse and human cancer cells, which inhibited the growth of these cells and led to the release of lactate dehydrogenase and galactin-3 from them. In addition to interacting with galactin-3, some other potential anticancer effects of modified pectins have been reported in both in vitro and in vivo conditions^18^. Low molecular weight citrus fractionated pectin powder (FPP) produced by autoclave hydrolysis or a combination of heating and enzymatic hydrolysis also induces androgen and androgen-independent prostate cancer cell apoptosis. MCPs produced by enzymatic degradation of pectasol, induced apoptosis in prostate cancer cells and increased the doubling time of prostate-specific antigen (PSA) in patients undergoing various treatments used to treat prostate cancer. Enzymatically hydrolyzed citrus pectin has also been shown to have clinical benefits, improved quality of life, and reduced pain in patients with advanced tumors in various types of cancer^19^.

Pectins can be degraded to pectic oligosaccharides (POS) by various degradation methods such as enzymatic hydrolysis, acid hydrolysis, hydrothermal processing, high-pressure microfluidization, or photochemical reaction in TiO_2_-containing medium^10,20^. Enzymatic methods have significant advantages over other methods, such as: (1) the reaction is performed under mild operating conditions, (2) the hydrolysis medium does not cause corrosion, (3) no toxic or contaminating chemicals are used (4) hydrolysis is selective and affects only specific constituent units or bonds, as expected for an enzyme-catalyzed reaction; (5) reaction efficiency may be higher than that achievable by, chemical methods and (6) the formation of unwanted products is avoided^21^. Pectinolytic enzymes are classified into two main groups: (a) esterases including pectin methyl esterase, and (b) depolymerase including hydrolase and lyase^22^. Many fungal species can break down pectin by producing different pectinolytic enzymes. Studies have shown that *Alternaria sesami* produces pectinolytic enzymes including polygalacturonase transeliminases, pectin transeliminases, and polygalacturonases^23^. Elrod reported for the first time that the *Erwinia* bacterium could degrade pectin^23^. Zucker et al.^24^ and Chatterjee et al.^25^ demonstrated the production of induced and extracellular endopolygalacturonase by *Pseudomonas fluorescens* and *Erwinia*.

*Alcaligenes faecalis* as a member of *Alcaligenaceae* from *Betaproteobacteria* class has many applications in biotechnology and food and health industries. *Alcaligenes* spp. has been used in the production of plastic-like storage material, enzymes, polysaccharides, and also commercial production of amino acids as food additives^26^.

As a member of *Paenibacillaceae* from the *Bacilli* class, *Paenibacillus polymyxa* is involved in a variety range of physiological and biotechnological processes. Different strains of this bacteria can be used in nitrogen fixation, antibiotics production, and phosphorus solubilization in the soil. *P. polymyxa* is known to produce cell wall degrading enzymes with hydrolytic pathways. Different strains of *P. polymyxa* were reported to produce exopolysaccharides (EPS) which as a secondary metabolite have a wide range of applications in bioindustries^27^.

In the current study *A. faecalis,* AGS3 and *P. polymyxa* S4 were isolated and identified. The objective of this study was to investigate microbial degradation of pectin by pectinase of isolated bacterial isolates *A. faecalis* AGS3 and *P. polymyxa* S4 and for the first time identify products and then investigate the anticancer and antioxidative properties of obtained compounds.

## Results and discussion

### Identification of the isolates and assessment of the pectinase activity

**Figure 1:**
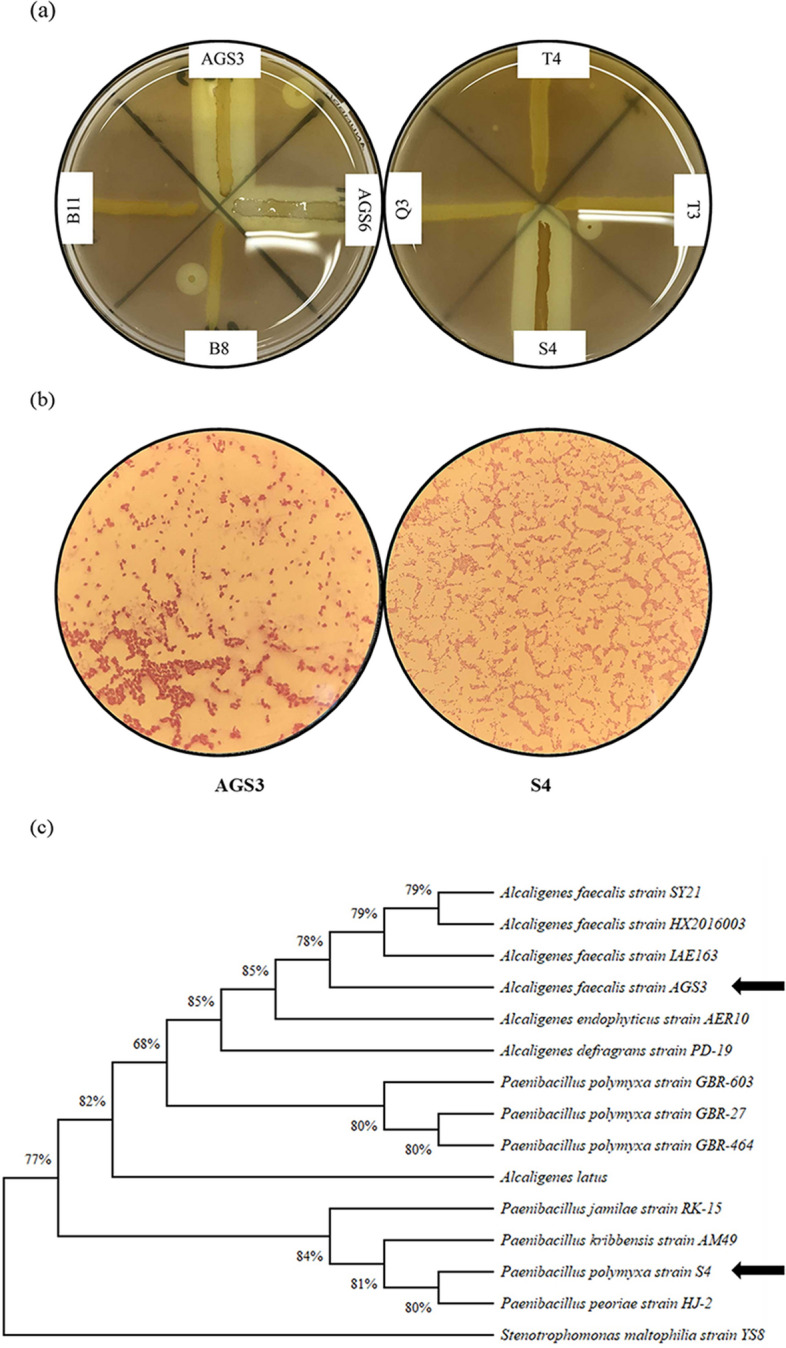
(**a**) Halo area around selected bacterial isolates, AGS3 and S4, showing pectinolytic activity by degradation of polygalacturonic acid (yellow halo areas). (**b**) Microscopic image of AGS3 and S4 bacterial isolates. (**c**) Phylogenetic relationships of *A. faecalis* isolate AGS3 and *P. polymyxa* isolate S4. The tree was constructed with MEGA 11 software using the Maximum-likelihood method with 1000 bootstraps.

Isolates AGS3, gram-negative, non-acid-fast, aerobic, rod-shaped, catalase-positive, oxidase-positive, citrate positive, and motile and S4 gram-positive, non-acid-fast, aerobic, rod-shaped, catalase-negative, oxidase negative, citrate negative, and not motile (Fig. 1b) were selected according to their clear halo area radius (Fig. 1a). Optimal growth conditions for both isolates were at 30 °C and pH 6.8–7.0. Results of phylogenetic analysis which was performed using the maximum-likelihood method showed that isolate AGS3 belongs to the *Proteobacteria* phylum, *Alcaligenaceae* family, and *Alcaligenes* genus, and S4 was a member of *Firmicutes* phylum, *Paenibacillaceae* family, and the genus of *Paenibacillus* (Fig. 1c). Further, BLAST analysis revealed that AGS3 and S4 isolates belonged to the species *Alcaligenes faecalis* and *Paenibacillus polymyxa* respectively. Henceforth, the sequence of isolates *A. faecalis* AGS3 (MZ093052) and *P. polymyxa* S4 (MZ596260) was submitted to GenBank, The National Center for Biotechnology Information (NCBI).

*Alcaligenes faecalis* is best known for anaerobic respiration with nitrite, and degradation of microbial reserve polymers such as poly-(3-hydroxybutyrate) (PHB). *A. faecalis* has also been used in the production of nonstandard amino acids. Although *A. faecalis* is recognized to have a variety of hydrolytic enzymes that can be used in the biodegradation of plant wastes, the study on its potential needs more progress^26^.

*Paenibacillus polymyxa* is recognized for producing a wide variety of secondary metabolites, which enables it to resist various environmental stresses and makes it an auspicious biotechnological agent in agriculture and industrial processes. *P. polymyxa* has the nitrogen-fixing ability, as well as the production of plant growth-regulating factors, hydrolytic enzymes, and antibiotic compounds. *P. polymyxa* strains are known for producing several hydrolytic enzymes including proteases, β-1,3-glucanases, cellulases, xylanase, lipase, amylase, chitinases, and pectinase. Therefore, various studies investigated *P. polymyxa* strains’ capability in waste management and wastewater treatment. However, compounds produced by the hydrolytic activity of *P. polymyxa* enzymes have not been yet identified and need more investigation^27^.

**Figure 2:**
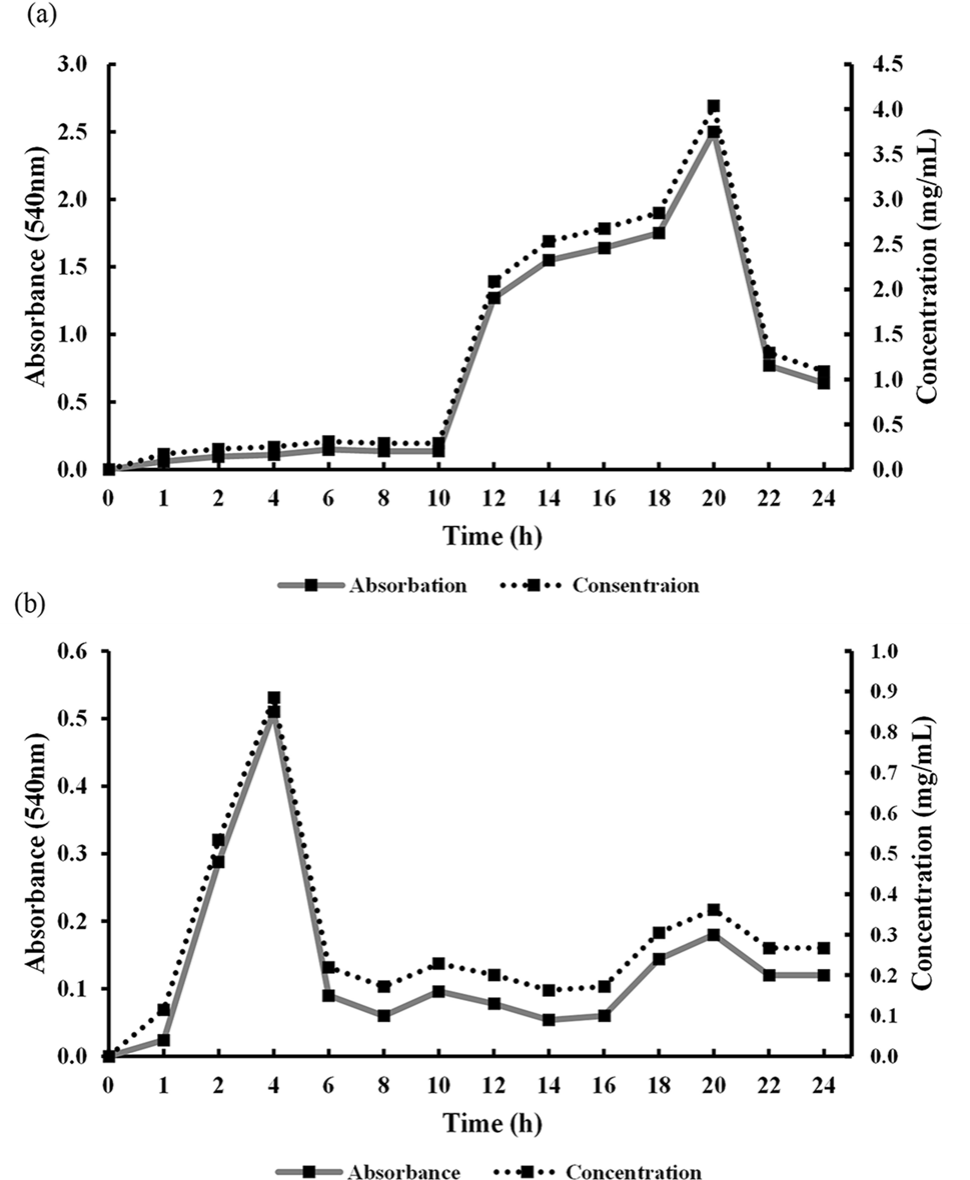
Monitoring amount of pectic oligosaccharide released from (**a**) *A. faecalis* AGS3 isolate, and (**b**) *P. polymyxa* S4 isolate, in pectin culture medium—30 °C 180 rpm.

Pectin degradation assay was employed using DNS reagent to determine the number of pectic oligo-saccharides (POS) produced by pectinase activity of *A. faecalis* AGS3 and *P. polymyxa* S4 isolates. The results of determination of the highest yield of POS release showed that *A. faecalis* AGS3 (Fig. 2a) and *P. polymyxa* S4 (Fig. 2b) isolates have their maximum concentration of released sugars after 20 h and 4 h incubation at 30 °C, 180 rpm respectively.

In the end, obtained POSs were lyophilized and stored for further analysis.

### Thin-layer chromatography and LC–ESI–MS of obtained products

**Figure 3:**
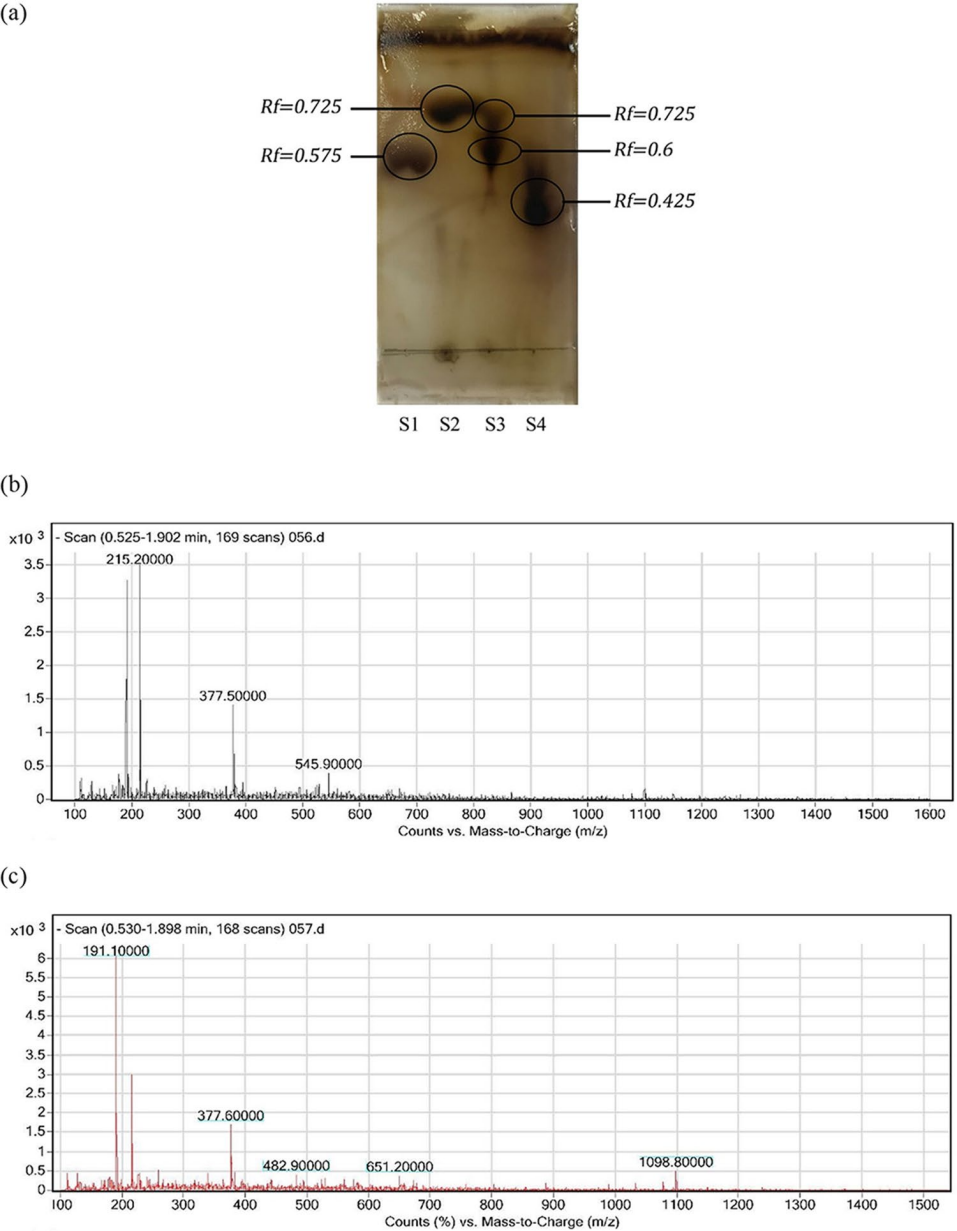
(**a**) TLC assessment patterns of samples obtained from the pectinolytic activity of isolates, S1: standard solution of glucose, S2: pectic oligosaccharide obtained from *A. faecalis* AGS3, S3: pectic oligosaccharide obtained from *P. polymyxa* S4, S4: standard solution of mono-galacturonic acid; LC–ESI–MS mass spectrums of samples obtained from pectin degradation by (**b**) *A. faecalis* AGS3, and (**c**) *P. polymyxa* S4.

Thin-layer chromatography (TLC) was performed to verify the degradation of pectin by the isolates. TLC patterns confirmed the degradation of pectin to POS in both isolates (Fig. 3a). Further analysis of produced oligo-galacturonic acids was implemented by LC–ESI–MS of lyophilized samples. The LC–ESI–MS mass spectrums of *A. faecalis* AGS3 and *P. polymyxa* S4 isolates products fractions showed that different types of oligo-galacturonic acids were present in enzymatic products of pectin degradation in both isolates (Fig. 3b, c, Table 1). In the end, forms of mono galacturonic acid and unsaturated mono, di, tri, and penta galacturonic acid were identified. There are different types of pectinolytic enzymes. Among them reaction conducted by pectin lyase, produces unsaturated oligo-galacturonates, therefore considering products obtained in this study, it is assumed that pectinolytic activity of *A. faecalis* AGS3 and *P. polymyxa* S4 isolates are due to pectin lyase enzyme.

**Table 1:** Identified compounds obtained from the pectinolytic activity of *A. faecalis* AGS3 and *P. polymyxa* S4 by analysis of their LC–ESI–MS spectrums.

Compound	*P. polymyxa* S4	*A. faecalis* AGS3	m/z
mono^1^, unsat^2^	+	+	191
mono, unsat, H^+^	+	+	193
mono, H^+^	+	+	195
d-Galacturonic acid Anhydro	+	+	199
mono, unsat, Na^+^	+	+	215
mono, Na^+^	+	+	218
d-Galacturonic acid, l-aceric acid	+	+	377
di^3^, unsat, H^+^, two acetyls, boron	+	−	482
di^3^, unsat, H^+^, tri acetyl, two methyl	+	+	545
tri^4^, one methyl, Na	+	+	583
tri, tri methyl, one unsat, H	+	+	587
tri, one methyl, one acetyl, one unsat, H	+	+	601
tri, one acetyl + ammonium	+	+	607
tri, one methyl, one acetyl, Na	+	+	625
tri, unsat, two Cl, H^+^	+	−	651
tri, unsat, two Cl	+	−	671
Penta^5^, unsat, tri acetyl group	+	+	1098

+ represents the presence and, − represents absence.

^1^Monogalacturonic acid,^2^unsaturated,^3^digalacturonic acid,^4^trigalacturonic acid,^5^pentagalacturonic acid.

In general, most reported pectinases belong to the fungi such as *Aspergillus*, *Alternaria*, and *Penicillium*^28–31^. Nevertheless, there have been reports showing various types of pectinolytic enzymes in the bacteria; for instance, *Acinetobacter guillouiae*, *Kosakonia sacchari*, and *Bacillus vallismortis* have been reported to have polygalacturonases. Pectin and pectate lyases have been proven to be present in *Streptomyces, Actinomycetes*, *Pseudomonas,* and *Bacillus* species^31,32^. There are some endophytic pathogens such as *Xanthomonas compestris*, *Ervinia chrysanthemi*, *Colletotrichum lindemuthianum*, *Pseudomonas siringea*, and *Phytophthora capsici* which are reported to have pectinolytic activity^31^. Studies suggest that some enteric pathogens including *Salmonella* and *Escherichia coli*, despite their lack of pectinolytic enzymes, are capable of using oligomers that result from pectin degradation^10,31^. We believe that this study is the first to identify compounds obtained from the pectinolytic activity of *A. faecalis* and *P. polymyxa*.

### Determination of antioxidative effects of POS

**Figure 4:**
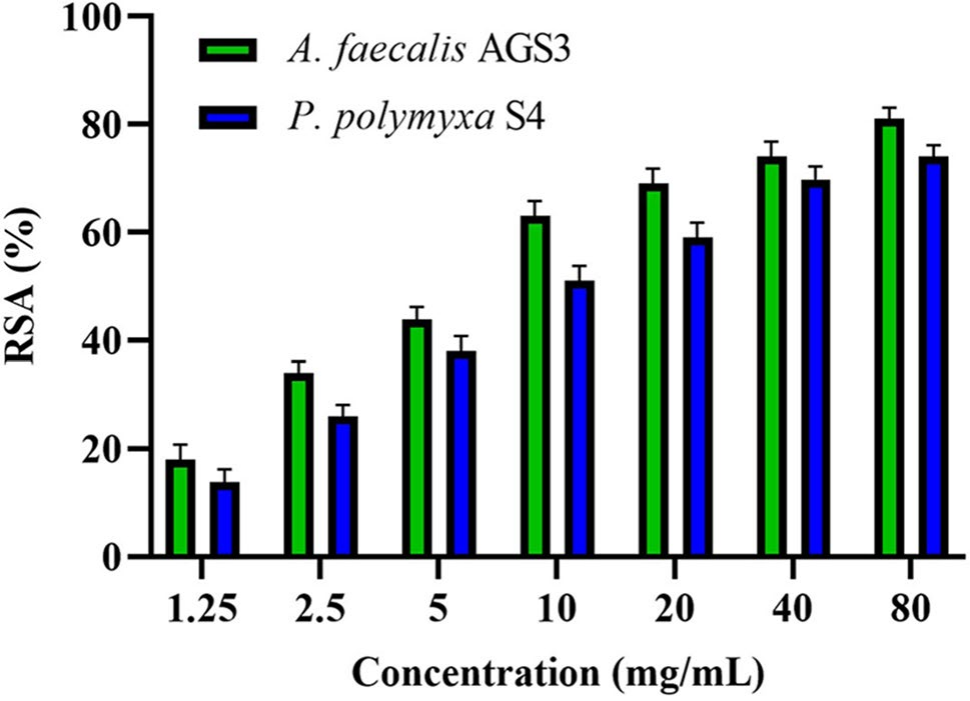
Assessment of antioxidant properties of oligosaccharides obtained from pectin degradation by DPPH reagent.

Radical scavenging activity (RSA) of obtained POSs, was tested by the DPPH reagent. The results for a range of concentrations from 1.25 to 80 mg/mL revealed that the antioxidative effects of POSs get higher by raising their concentrations. RSA of POS obtained from *A. faecalis* AGS3 isolate ranged from 18 to 81% for 1.25 to 80 mg/mL concentrations, and the results for *P. polymyxa* showed a range of RSA from 13 to 74% for the same concentrations (Fig. 4).

Oxidative stress is a concept that is associated with the loss of balance between pro-oxidants and antioxidants and is related to the physiology of common diseases. Antioxidants have their role in neutralizing reactive forms of oxygen with a negative impact on living cells^33^. Yeung et al.^34^ used the Fenton reaction to hydrolyze okra pectin and found that the antioxidant activity of obtained POS is concentration-dependent. The results of the DPPH assay of POS obtained by enzymatic hydrolyzation *Streptomyces hydrogenases* YAM1 showed that RSA increases in higher concentrations. Hosseini Abari et al.^10^ also demonstrated that enzymatically modified pectin has more antioxidative activity compared to untreated pectic polysaccharides. Similar studies corroborate the dose-dependency of POSs antioxidative activity. In this study, as shown in Fig. 6, regarding an increment of antioxidant properties of samples by concentration, the highest RSA was reported in 80 mg/mL of samples obtained from *A. faecalis* AGS3 and *P. polymyxa* S4. In 20 mg/mL of POS, results were 59% for *P. polymyxa* S4 and 69% for *A. faecalis* AGS3, which showed a 20–30% raise in RSA, compared to polygalacturonic acids as mentioned in Hosseini Abari et al. study^10^. These results show a significant increment in RSA of POS in comparison to the results of the antioxidative activity of pectic polysaccharides from previous studies. This is the first report of RSA in products of *A. faecalis* and *P. polymyxa* species.

### Determination of the anticancer effects on MCF‑7 human breast cancer cells.

**Figure 5:**
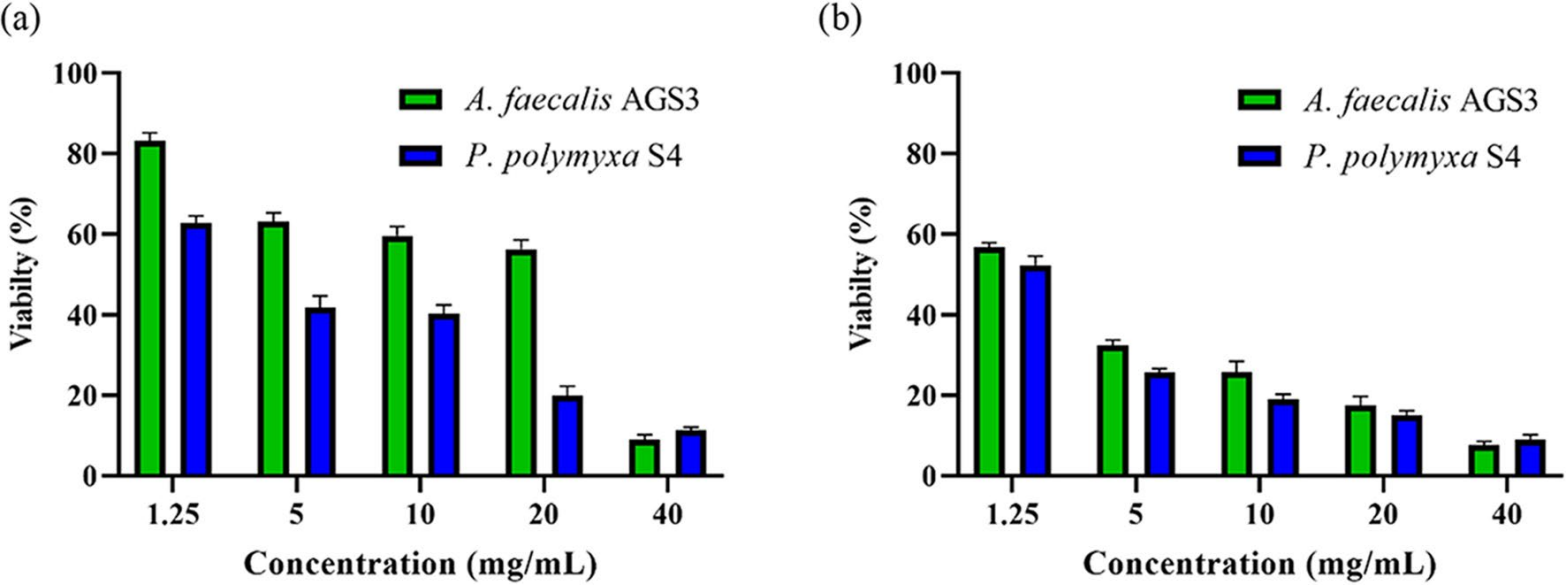
MTT assessment of MCF-7 cells after treatment for (**a**) 24 h and (**b**) 48 h.

An assessment carried out by MTT assay and flowcytometry showed significant anticancer activity on MCF-7 cells for POS obtained from apple waste using pectinolytic enzymes of *P. polymyxa* S4 and *A. faecalis* AGS3 isolates. As mentioned in Fig. 5, results of the MTT assay showed a maximum cell viability reduction at 40 mg/mL of POSs obtained from *A. faecalis* AGS3 and *P. polymyxa* S4 with 93% and 91% respectively after 48 h incubation. Minimum cell viability reduction was obtained at 1.25 mg/mL of POS after 24 h of treatment with 17% and 37% for *A. faecalis* AGS3 and *P. polymyxa* S4, respectively.

**Figure 6:**
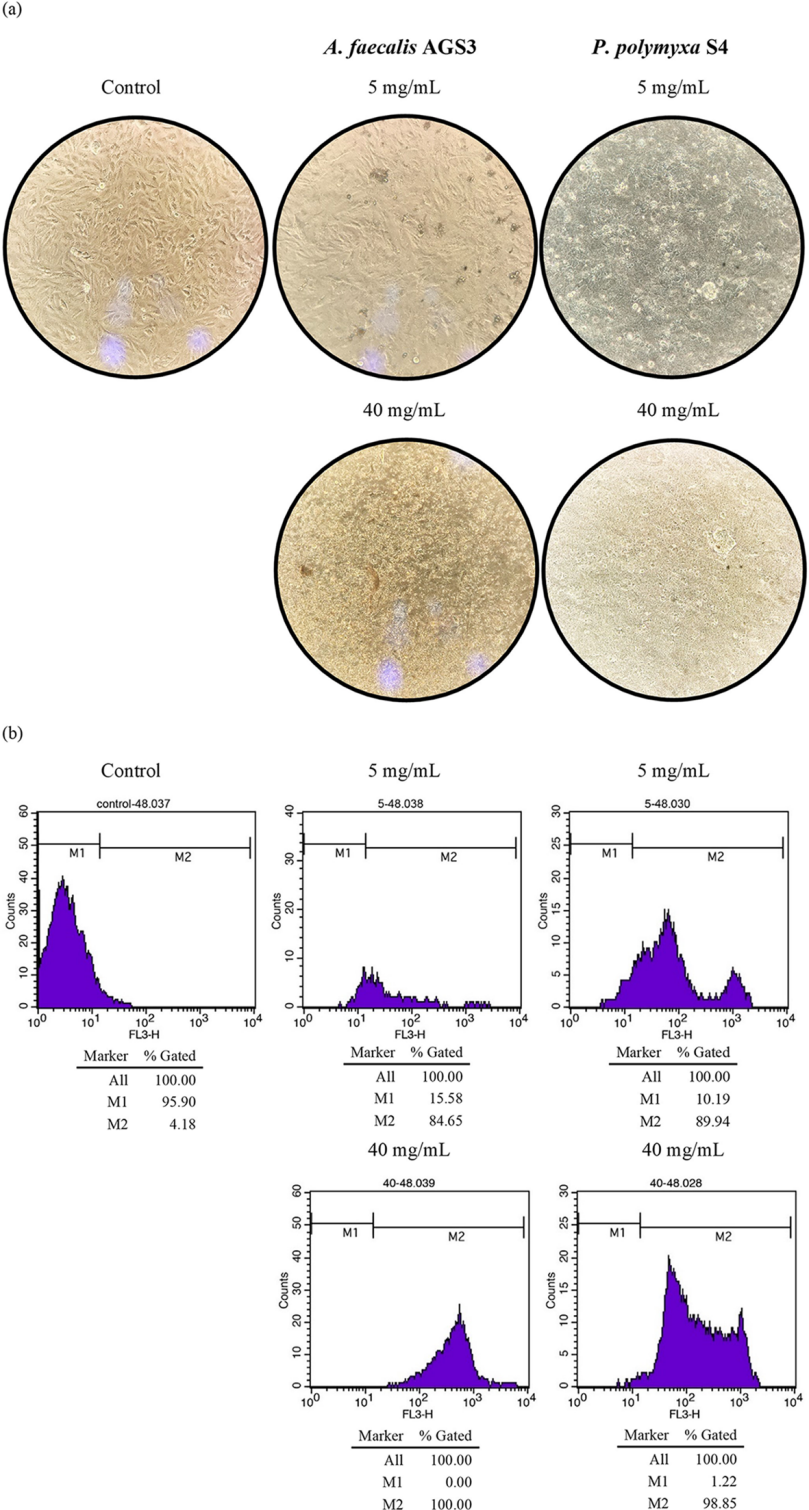
Cell viability results of MCF-7 after 48 h treatment with 5 and 40 mg/mL of POS obtained *A. faecalis* AGS3 and *P. polymyxa* S4, where (**a**) shows morphological aspects of treated cells in comparison to control (untreated) cells and (**b**) demonstrates flow cytometric analysis of treated cells, compared to control (untreated) cells.

Likewise, results of flow cytometric analysis at 5 and 40 mg/mL of obtained POSs after 48 h incubation demonstrated induction of apoptosis, with 84% and 100% for *A. faecalis* AGS3, and 90% and 98% for *P. polymyxa* S4 (Fig. 6b). In Fig. 6b M1 zone represents the distribution of living cells, not stained by the PI reagent, and the M2 zone demonstrates dead cells, stained by the PI reagent. As is visible in Fig. 6a, treated cells also were subject to morphological changes.

Cancer as one of the significant health problems in the world, has numerous physiological and biochemical inducers called carcinogens. The majority of synthetic drugs used in chemotropic treatments due to their side effects on non-cancerous cells and causing drug resistance of cancerous cells can originate more problems for patients, therefore using natural compounds with high anticancer properties has become a superior solution^10,13,35^. Studies have shown that enzymatically hydrolyzed citrus pectin fragments can affect the progression of proliferative prostate cancer by reducing serum PSA by 50% after 14 months of treatment. It has been proven that enzymatically treated citrus and apple pectins can inhibit growth and induce apoptosis in human intestinal cancer cells^19^. Low molecular weight modified apple pectin inhibits the cell cycle in colorectal tumors in human colorectal cancer cells (HT-29) in vitro and colitis-associated colorectal cancer in mice^36^. The same low molecular weight apple pectin reduced the risk of colon cancer tumors in mice and was reported to bind to galactin-3^18^. Examination of the monosaccharide composition showed that galacturonic acid was the major component of the modified pectin structure and only small amounts of galactose were found in it, however, the monosaccharide composition is similar to that of pectasol monosaccharides. While MCP may not be rich in galactooligo-saccharides, it can be selectively absorbed in the small intestine compared to oligo-galacturonic acids^37^. In the study of Delphi et al. on MDA-MB-231 cancer cells, treatment with POS induced apoptosis and reduced survival of cancer cells^38^. The results of measuring the effect of pectic oligosaccharides and pectic polysaccharides on the HT29 cancer cell line by Li et al.^39^ showed that POSs are more effective in inhibiting cancerous cells in much lower concentrations than pectin. Hosseini Abari et al.^10^ demonstrated 92% apoptosis of MCF-7 cell line after 24 h treatment with 20 mg/mL POS which was significantly higher than pectin’s effect in the same concentration. Supporting previous results, our study obtained oligosaccharides that showed significant anticancer properties for both *P. polymyxa* S4 and *A. faecalis* AGS3 samples. To the best of our knowledge, there are no previous reports of anti-tumor properties for *P. polymyxa* and *A. faecalis* species products.

### Determination of the cytotoxicity effects of POS on L-929 mouse fibroblast cells

**Figure 7:**
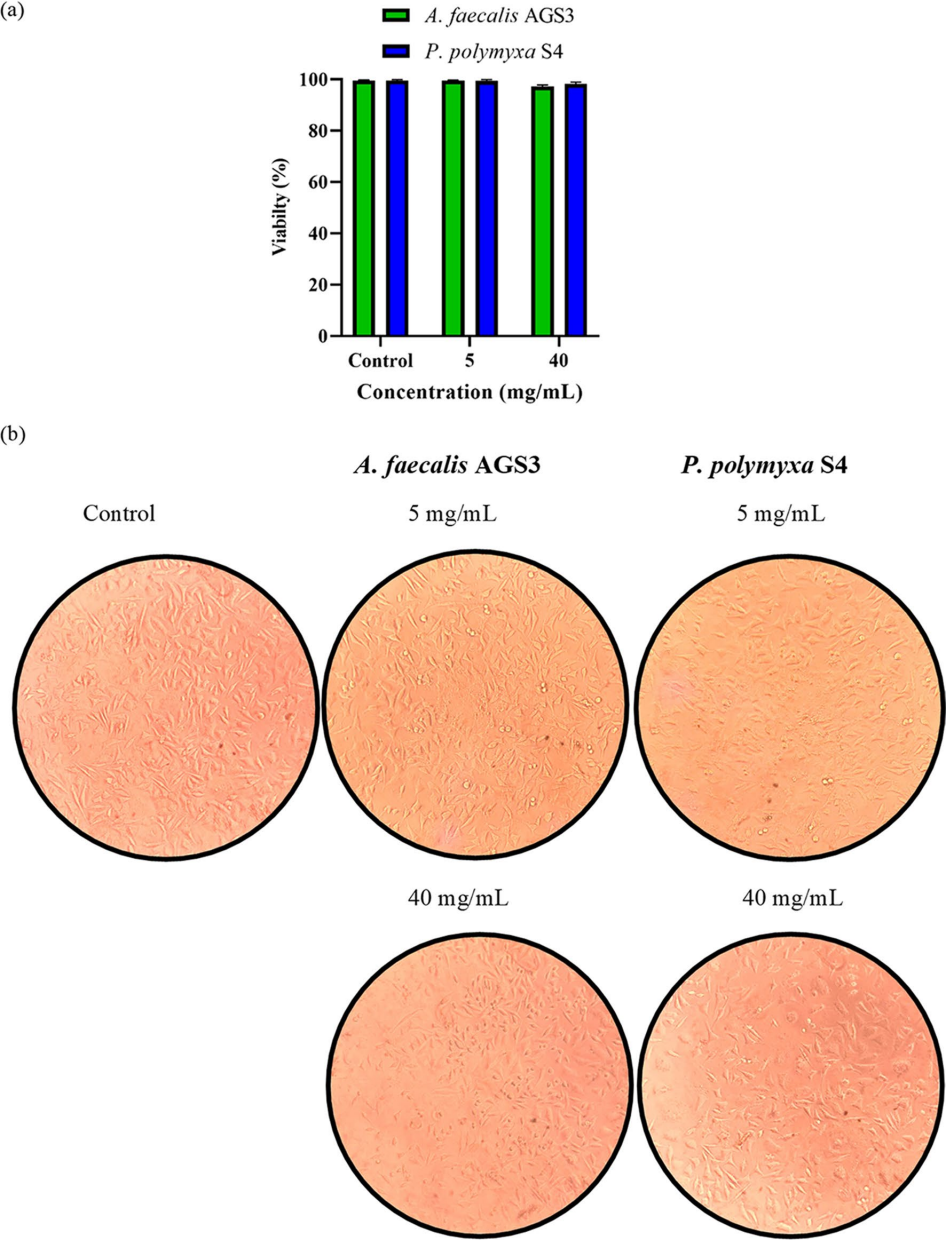
Cytotoxicity assessment of obtained POSs on L-929 cells after 48 h incubation. (**a**) MTT assay results of treated cells showed no significant cytotoxicity in comparison to control. (**b**) Microscopic analysis of treated cells showed no change in morphology of cells compared to control.

Cytotoxicity effects of obtained POSs on L-929 cells were determined after 48 h incubation by MTT assay. L-929 cells were treated with 5 and 40 mg/mL of obtained products and untreated cells were used as control. As mentioned in Fig. 7a, the analysis showed no significant toxicity for *A. faecalis* AGS3 with less than 3% death, and *P. polymyxa* S4 with about 2% death. Morphology of treated cells as shown in Fig. 7b confirmed the results of the MTT assay.

Cytotoxicity of drugs used in treating cancer on non-cancerous cells is a major concern in chemotropic treatments. Opposing to previous results from Delphi et al. which showed toxicity on high concentrations of POS on HUVEC cells, in the current study after 48 h treatment of L-929 cells with 40 mg/mL of POSs, no significant toxicity was observed^38^. According to our information, no other study investigated the cytotoxicity of POS obtained from *A. faecalis* and *P. polymyxa*.

## Conclusion

As a result of this study, two new bacterial isolates, *Alcaligenes faecalis* AGS3 and *Paenibacillus polymyxa* S4 were isolated and identified. Mentioned isolates were able to degrade pectin to a range of unsaturated pectic oligo-saccharides. In this study for the first time, the bioactivity of compounds obtained from *A. faecalis* and *P. polymyxa* species pectinolytic activity was investigated, and results showed significantly higher antioxidant and anticancer properties despite being non-toxic to living cells. The exquisite bioactivity of obtained compounds, alongside the ability to eliminate fruit waste from the environment, makes them a great economic and environmental solution to a growing problem in the world.

## Methods

### Extraction of pectin from apple waste

Waste from the Golden Delicious cultivar of apple (*Malus domestica*) was used in this study. Mentioned waste was purchased from the local fruit market of Bahar fruit shop, Isfahan, Isfahan Province, Iran. The apples were peeled off and chopped into 3 mm cubes then 30 g of the chopped apple waste was added to a solution consisting of lemon juice (12.5 mL), citric acid (0.1 g), and 150 mL distilled water. The mixture was boiled on the heater for 30 min and then filtered through a cotton cloth. After cooling down to room temperature, 30 mL of 96% ethylic alcohol was added to the filtered solution and then put at 4 °C for an hour to gain gelling form. The supernatant of the mixture was discarded after centrifuging for 10 min at 800 g. In the end, the sediment which contained the extracted pectin was freeze-dried^40^.

### Isolation and identification of pectin degrading bacteria

Soil samples were gathered from 12 different areas as the isolation source. Samples were dissolved and well mixed for an hour in Ringer's Solution, then cultivated in nutrient agar medium containing 0.25 mL of 1% clotrimazole in 100 mL distilled water. Selected isolates then were cultivated in pectin agar medium containing extracted pectin (0.5 g), yeast extract (0.1 g), peptone from casein (0.5 g), CaCl_2_ (0.2 g), NaCl (0.2 g), and agar (1.5 g) in 100 mL distilled water^41^. After 24 h at 30 °C, pectin-degrading colonies were differentiated by measurement of their clear halo area as a result of adding 1% I/KI reagent solution to the plates^42^. Isolates AGS3 from garden soil, and S4 from forest soil were selected and identified. The universal primer pairs, 27F (5′-AGAGTTTGATCCTGGCTCAG-3′) and 1492R (5′-TACGGTTACCTTGTTACGACTT-3′) were used as forward and reverse primers respectively to amplify 16S rRNA gene and then the sequencing procedure was performed by Bio Magic Gene (BMG) Company, China. The sequences were submitted to GenBank, NCBI, and evolutionary relationship history was investigated using the maximum likelihood tree method in MEGA 11 software. The 16S rDNA sequence of *Streptomyces maltophilia* strain YS4 was used as an outgroup (MT071635.1).

### Pectin degradation assay

Pectin degradation was determined, using the quantitative analysis of reducing sugars by 3, 5-dinitrosalicylic acid (DNS method). After inoculation of bacterial isolates to the pectin broth medium containing extracted pectin (0.5 g), yeast extract (0.1 g), peptone from casein (0.5 g), CaCl2 (0.2 g), and NaCl (0.2 g) in 100 mL distilled water, 4 mL of bacterial medium was extracted in sterile conditions every two hours. Then bacterial mass was separated from media by centrifuging at 2400 g for 5 min. A 1 mL of DNS reagent was added to a screw-capped tube containing culture supernatant, and reaction tubes were boiled for 5 min and cooled at room temperature. A 1 mL of 0.5% sodium sulfate was added to test tubes to obtain a stable color and the absorbance values were read at 540 nm^43^.

### Analysis of obtained oligo‑galacturonic acids

#### Thin-layer chromatography

Thin-layer chromatography (TLC) was used to detect products of pectin degradation. A 1.5 μL of the culture supernatants as a sample, 1 mM solution of mono-galacturonic acids and a 1 mM solution of Glucose as standard solutions (purchased from Sigma) were spotted on silica gel 60 F254 (Merck). Chromatography was performed thrice in n-butanol/acetic acid/water (2:1:2) as a mobile phase. Visualization of the dried spots on silica gel was performed by spraying orcinol/sulfuric acid reagent (8 mg orcinol in 10 mL of 70% sulfuric acid). Then plates were heated at 100 °C for 10 min^32^.

#### Liquid chromatography-electrospray ionization-mass spectrometry (LC–ESI–MS).

The obtained products of pectin degradation were analyzed and identified by LC–ESI–MS. A 2 mg of each sample was dissolved in 100 μL distilled water and the soluble fraction of the samples was investigated. The investigation was performed isocratically by a mixture of water and acetonitrile (90:30) as a mobile phase and the flow rate was set to 0.3 mL/min. An Agilent 1100 series LC system consisting of a quaternary delivery pump, a thermostated column compartment, a degasser (Agilent Technologies, Germany), and a Rheodyne 7725i manual injector valve with a 20 µL sample loop (Cotati, CA, USA) was used to prepare a sample and the mass spectrometry was carried out with Agilent 6410 Triple Quadrupole mass spectrometer (Agilent Technologies, Palo Alto, CA, USA) which was run by Agilent MassHunter Workstation B.01.03. Ionization was attained using electrospray ionization (ESI) in the negative mode with the capillary voltage of 4000 V. Nitrogen was used as nebulizer gas with a nebulizer pressure of 40 psi and source temperature of 100 °C. Nitrogen was heated to 300 °C and delivered at a flow rate of 10 L/min. The fragment voltage for the samples was 280 V and the dwell time was 200 ms. The analyte was detected using scan mode^32^.

#### Determination of antioxidative effects of the pectic oligo‑saccharides (POS)

The antioxidative effects of obtained pectic oligo-saccharides were assessed at various concentrations ranging from 1.25 to 80 mg/mL. A 0.5 mL of POS was added to 2 mL of 0.2 mM methanolic solution of 2,2-diphenyl-1-picrylhydrazyl (DPPH). The reaction tubes were placed for 30 min at 25 °C in darkness. Afterward, the absorbance of the samples was examined at 517 nm. Control of the reaction was 0.2 mM DPPH and 60% ethanol was used as blank. Based on measured absorbance, radical scavenging activity (RSA) was calculated by Eq. (1)^44^:

1 $$ {\text{RSA }}\left( \% \right)\, = \,\left[ {\left( {{\text{Abs control}}{-}{\text{Abs sample}}} \right)/\left( {\text{Abs control}} \right)} \right]\, \times \,{1}00 $$

#### Determination of the anticancer effects of POS on MCF‑7 human breast cancer cells

MCF-7 cell line of human breast cancer (acquired from the University of Isfahan Cell Bank) was cultivated in COM medium made of Dulbecco’s modified Eagle’s minimum media (DMEM; BioIdea), with 10% fetal bovine serum (BioIdea) and 1% penicillin–streptomycin solution (Sigma, USA)^45^.

#### MTT assay

MTT ((3-(4,5-dimethylthiazol-2-yl)-2,5-diphenyl tetrazolium bromide) assay was used to analyze the cell viability. The cells were plated in 96 well culture plates at a density of 10,000 cells per well and incubated in a CO_2_ incubator at 37 °C in 5% CO_2_ and 95% humidity for 24 h. The next day the cells were washed with phosphate-buffered saline (PBS, pH 7), treated with 5 and 40 mg/mL concentration of pectin, and obtained POS for 24 and 48 h. Further, at respective time points, 20 μL MTT solution (5 mg/mL) was added to wells, and cells were incubated in a CO_2_ incubator in the dark for 4 h. The medium was removed and Formazan crystals formed by the cells were dissolved using 100 µl of dimethyl sulfoxide (DMSO). The cell viability was measured using a multi-mode reader at 570 nm and calculated as Eq. (2)^46^:

2 $$ {\text{Cell viability }}\left( \% \right)\, = \;\left[ {\left( {{\text{Abs of treated cells}}/{\text{Abs of untreated cells}}} \right)\, \times \,{1}00} \right]. $$

#### Flow cytometry

The cells were harvested onto 24 well culture plates at a density of 100,000 cells per well and incubated in a CO_2_ incubator for 24 h. The cells were washed with PBS and treated with 5 and 40 mg/mL concentrations of obtained POS for 24 and 48 h. Henceforth, propidium iodide (PI, Sigma) was used for the detection of immortalized cells. The cells were assessed using Flow Cytometer (Becton Dickinson FACS Calibur) and the distribution of cells was analyzed using CellQuest software^47^.

#### Determination of the cytotoxicity effects of POS on L-929 mouse fibroblast cells

L-929 mouse fibroblast cells were cultivated in COM medium and plated to 96 well culture plates. After 24 h cells were treated using 5 and 40 mg/mL concentration of obtained POS for 24 and 48 h. Afterward, the MTT assay as mentioned was performed to analyze the cytotoxicity effects of obtained compounds^46^.

## Supplementary Information

Supplementary Information.

## Data Availability

All data analyzed during this study are included in this published article and its Supplementary Materials files.
