# Supplementary material for: Investigation of bioactivity of unsaturated oligo‑galacturonic acids produced from apple waste by Alcaligenes faecalis AGS3 and Paenibacillus polymyxa S4 Pectinases
Source: Sci Rep. 2022 Sep 22;12:15830. doi: 10.1038/s41598-022-20011-2 (PMC9500027; doi:10.1038/s41598-022-20011-2)
Supplement: Supplementary file 1 — Supplementary Information. [file 41598_2022_20011_MOESM1_ESM.docx]

**Supplementary Information:**

In order to choose isolates with proper pectinolytic activity, 80 bacterial colonies were isolated from different sources. Among them 52 isolates had the ability to grow on pectin agar medium. Afterward halo area around those isolates was investigated and 32 of them had halo area caused by pectin hydrolyzing enzyme. The Table describes each codename’s location, source, type of source which were used to classify isolates.

**Table.** Description of codename of bacterial isolates used in the current study.

| Source | Type of source | Isolation location | Codename |
| --- | --- | --- | --- |
| Fruit garden | Soil | Hashtgerd, Alborz, Iran | AGS |
| Salt Lake | Soil | Urmia, West Azerbaijan, Iran | OLS |
| Fruit garden | Soil | Isfahan, Isfahan, Iran | B |
| Fruit garden | Soil | Zarandieh, Markazi, Iran | PVS |
| Fruit garden | Soil | Dezful, Khuzestan, Iran | DOS |
| Fruit garden | Soil | Dezful, Khuzestan, Iran | DSOS |
| Forest | Soil | Savadkuh, Mazandaran, Iran | S |
| Forest | Soil | Tabriz, East Azerbaijan, Iran | T |
| Forest | Soil | Qaleh roudkhan, Gilan, Iran | Q |
